# An optically-guided cochlear implant sheath for real-time monitoring of electrode insertion into the human cochlea

**DOI:** 10.1038/s41598-022-23653-4

**Published:** 2022-11-10

**Authors:** Anastasiya Starovoyt, Bryden C. Quirk, Tristan Putzeys, Greet Kerckhofs, Johan Nuyts, Jan Wouters, Robert A. McLaughlin, Nicolas Verhaert

**Affiliations:** 1 Department of Neurosciences, ExpORL, KU Leuven, 3000 Leuven, Belgium; 2 Department of Neurosciences, Leuven Brain Institute, KU Leuven, 3000 Leuven, Belgium; 3 Australian Research Council Centre of Excellence for Nanoscale BioPhotonics, Faculty of Health and Medical Sciences, The University of Adelaide, Adelaide, SA 5005 Australia; 4 Institute for Photonics and Advanced Sensing, The University of Adelaide, Adelaide, SA 5005 Australia; 5 Laboratory for Soft Matter and Biophysics, Department of Physics and Astronomy, KU Leuven, 3000 Leuven, Belgium; 6 Biomechanics Laboratory, Institute of Mechanics, Materials, and Civil Engineering, UCLouvain, 1348 Louvain-La-Neuve, Belgium; 7 Department of Materials Science and Engineering, KU Leuven, 3000 Leuven, Belgium; 8 Institute of Experimental and Clinical Research, UCLouvain, 1200 Woluwé-Saint-Lambert, Belgium; 9 Prometheus, Division of Skeletal Tissue Engineering, KU Leuven, 3000 Leuven, Belgium; 10 Department of Imaging and Pathology, Division of Nuclear Medicine, KU Leuven, 3000 Leuven, Belgium; 11 Nuclear Medicine and Molecular Imaging, Medical Imaging Research Center, 3000 Leuven, Belgium; 12 School of Engineering, University of Western Australia, Perth, WA 6009 Australia; 13 Department of Otorhinolaryngology, Head and Neck Surgery, University Hospitals of Leuven, 3000 Leuven, Belgium

**Keywords:** Translational research, Cochlea, Imaging and sensing

## Abstract

In cochlear implant surgery, insertion of perimodiolar electrode arrays into the scala tympani can be complicated by trauma or even accidental translocation of the electrode array within the cochlea. In patients with partial hearing loss, cochlear trauma can not only negatively affect implant performance, but also reduce residual hearing function. These events have been related to suboptimal positioning of the cochlear implant electrode array with respect to critical cochlear walls of the scala tympani (modiolar wall, osseous spiral lamina and basilar membrane). Currently, the position of the electrode array in relation to these walls cannot be assessed during the insertion and the surgeon depends on tactile feedback, which is unreliable and often comes too late. This study presents an image-guided cochlear implant device with an integrated, fiber-optic imaging probe that provides real-time feedback using optical coherence tomography during insertion into the human cochlea. This novel device enables the surgeon to accurately detect and identify the cochlear walls ahead and to adjust the insertion trajectory, avoiding collision and trauma. The functionality of this prototype has been demonstrated in a series of insertion experiments, conducted by experienced cochlear implant surgeons on fresh-frozen human cadaveric cochleae.

## Introduction

Over 5% of the world’s population suffers from disabling hearing loss, the majority of which concerns partial hearing loss^1^. Despite preserved ability to hear sounds, individuals with partial hearing loss often struggle with understanding speech, which severely affects their professional, social, emotional and cognitive well-being, and cannot be fully rehabilitated with hearing aids^2^. In the last two decades, cochlear implants (CI), which were originally developed to treat complete deafness, showed great potential for restoring speech understanding in patients with residual hearing^3^. However, the CI surgery poses a considerable risk for loss of residual hearing in this patient population. This frequent complication is predominantly caused by intraoperative trauma to the cochlea^4^, occurring in up to 32% of implantations^5^.

A CI consists of an external microphone and an electrode array, which is surgically inserted into the spiral-shaped cochlea to electrically stimulate the hearing nerve^6^. Ideally, the array is inserted into the scala tympani (ST) compartment, which is located adjacent to the auditory nerves inside the modiolus (the central cone of the cochlea)^6^. However, the cochlear anatomy varies greatly between and within individuals^7^. If the insertion trajectory does not perfectly match the shape of the ST, the electrode can traumatize the thin walls of the ST—the bony osseous spiral lamina (OSL) and the soft basilar membrane (BM)—or even translocate into one of the other two compartments—the scala vestibuli or the scala media^8^—resulting in a fistula. In particular, the thinner BM, on top of which resides the hearing epithelium, is very sensitive to mechanical trauma. Any trauma can lead to immediate hearing loss or result in chronic inflammation, fibrosis and delayed loss of residual hearing^4^. Therefore, risk-benefit consideration often favors late implantations in patients with residual hearing^9^. However, aside from the challenge of living with hearing disability, postponing of CI surgery leads to further deterioration of the hearing nerve in the absence of sensorial input, leaving less opportunity for improvement when CI surgery is finally performed^9^.

Electrode insertion trauma has been addressed to some degree by applying soft surgery techniques and by designing electrode arrays less prone to causing trauma. From the surgical point of view it is best to insert the electrode array at a very slow speed^10^ through the round window^11^, since this is the only part of the human cochlea not covered by bone. In addition, intraoperative administration of corticosteroids can help reduce the inflammatory response and subsequent residual hearing loss if low levels of trauma occur^4^.

For the electrode array design, a smaller diameter together with a soft, flexible electrode tip can help reduce the risk of trauma, whereas perimodiolar electrode positioning results in better implant performance due to closer proximity to the auditory nerves^12^. One design approach is to use a pre-coiled slim modiolar electrode array, which is loaded into a straight semi-flexible insertion device, called a sheath, during insertion^13^. This 5.5 mm long sheath acts as a guide catheter for insertion through the round window membrane or through a surgically drilled opening in the cochlear bone (a cochleostomy), and the entire length of the sheath is inserted into the ST. Once the sheath is inserted into its final position, the electrode array is pushed out through the sheath, whereby its pre-coiled shape naturally follows the inner curvature of the cochlea. Clinical studies showed that with this design, electrode translocations could be reduced to less than 6.6%^13–20^. The translocations that did occur typically happened in the proximal part of the cochlea, along the insertion trajectory of the sheath^18^. Unfortunately, this design resulted in tip fold-over in 2.0–7.7% of cases^14,15,17,19–23^, an example of which is clearly illustrated in the study of McJunkin et al.^22^. This appears to be related to suboptimal positioning of the sheath^13,21^. This fold-over phenomenon not only negatively affects the CI performance, but can also lead to cochlear trauma^24^. Thus, the correct positioning of the sheath is crucial to both reducing trauma and optimizing performance.

Since the human cochlea is entirely surrounded by bone, it is extremely challenging to assess the position of the sheath within the cochlea during the insertion. In current clinical practice, the surgeon primarily relies on tactile feedback and adjusts the insertion trajectory if increased resistance is experienced. However, increased resistance can only be perceived after the electrode has already touched the cochlear wall and possibly traumatized it. Furthermore, studies reported that electrode insertion trauma is often not accompanied by an increased resistance or mechanical forces, making it currently an unreliable parameter for insertion monitoring^25,26^. Intraoperative X-ray plain film fluoroscopy and transimpedance matrix measurements can help detect electrode fold-over, but do not prevent it and provide no information on the actual position of the electrode inside the ST with respect to the cochlear walls at risk for trauma^27^. Robotic electrode insertion based on preoperative imaging may reduce the risk of electrode translocation, but the value of the preoperative imaging is limited. In particular, the resolution of current preoperative imaging techniques, such as computed tomography (CT) and magnetic resonance imaging (MRI), is too low to visualize the thin cochlear walls, which is necessary for atraumatic trajectory planning^28^.

In recent years, optical coherence tomography (OCT) has shown potential to aid in surgical guidance. OCT is a non-invasive optical imaging modality commonly used in ophthalmology and cardiology. It uses reflections of non-ionizing, near-infrared light to acquire real-time, high-resolution 2D and 3D images of the tissue microarchitecture, typically at a resolution of 1–20 µm^29^. It is commonly deployed in an endoscopic or intravascular setting through the use of imaging probes that consist of a length of optical fiber to transmit the light deep inside the body, and a miniaturized lens to focus the light. Miniaturized fiber-optic OCT probes have been successfully used to perform real-time high-resolution imaging of a range of lumens, including airways, intestines and blood vessels^30^. Early work using side-facing, rotating fiber-optic probes has also shown the potential of OCT to acquire images of the cochlear lumen^31–33^. However, these probes are too large (minimal diameter: 0.35 mm)^33^ to be inserted simultaneously with the CI sheath (diameter: 0.65 mm)^13^ into the ST (proximal width: < 1 mm)^34^. Furthermore, the sheath cannot be rotated inside the cochlea, because it is designed to be inserted in one fixed orientation with respect to the modiolus^13^, which makes it incompatible with rotating fiber-optic probes.

In this study, we present a novel design for a CI sheath with an integrated, highly-miniaturized forward-facing OCT probe that can provide real-time feedback during insertion into the human cochlea. The diameter of our fixed, forward-facing probe is substantially smaller (0.125 mm) than the rotating, side-facing designs previously explored within the cochlea. We hypothesized that a forward-facing OCT probe would enable visualization of the cochlear lumen ahead of the sheath during insertion, permitting detection of the cochlear walls at risk of trauma, before they come into contact with the sheath. This would allow the surgeon to intra-operatively monitor the insertion and adjust the trajectory, if deemed necessary to avoid insertion trauma, based on the OCT feedback. We investigated the functionality of the optically-guided insertion sheath on fresh-frozen human cadaveric cochleae, and validated the results against contrast-enhanced microCT (CECT) imaging. In the remainder of the paper, we refer to this prototype device as the optically-guided sheath.

## Results

### The optically-guided sheath

**Figure 1:**
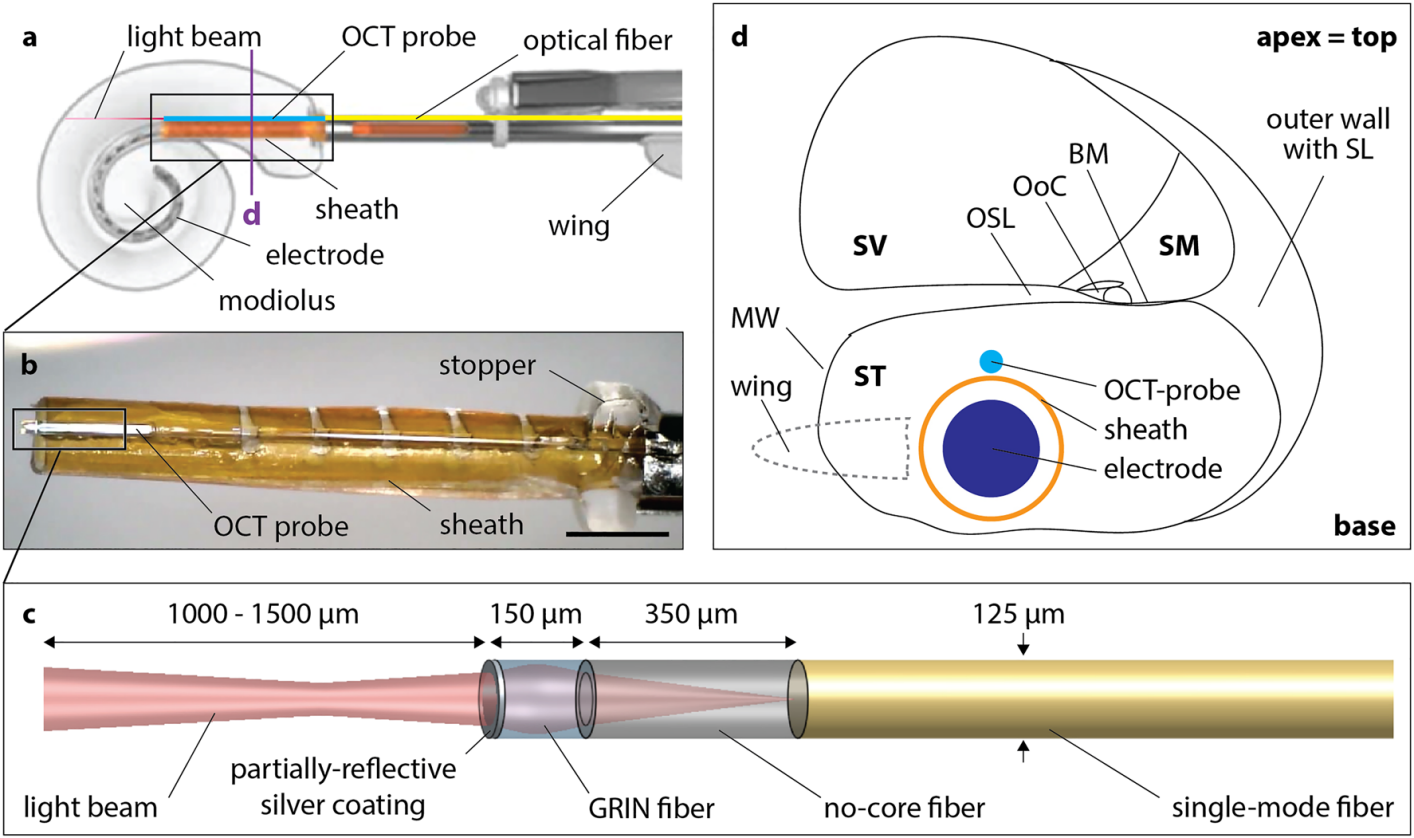
Design and intracochlear position of the optically-guided sheath. (**a**) A schematic shows the optically-guided sheath and the electrode within the cochlear spiral, whereby the electrode is positioned adjacent to the modiolus (original schematic was adapted with permission, courtesy of Cochlear Limited). Note the position of the sheath wing, pointing towards the modiolus. The purple line indicates the position of the schematic cross-section depicted in (**d**) with respect to the cochlear spiral. (**b**) Microscopic photograph of the optically-guided sheath. (**c**) Schematic of the OCT probe together with its beam path. (**d**) Schematic of the optically-guided sheath inside the ST and its relation with respect to the cochlear walls and the other compartments. The orientation of the sheath wing, located outside the cochlea, is indicated. *BM* basilar membrane, *GRIN fiber* graded-index fiber, *MW* modiolar wall, *OoC* Organ of Corti, containing the hearing epithelium, *OSL* osseous spiral lamina, *SL* spiral ligament, *ST* scala tympani, *SV* scala vestibuli. Scale bar: 1 mm.

During the insertion of a CI sheath into the ST, three cochlear walls can be at risk of trauma: the modiolar wall (MW), the OSL and the BM. All of these walls are positioned on the apical—‘top’—side of the ST. The base of the ST consists of thick, cortical bone, which is not at risk of trauma, and the intrinsic design of the sheath avoids contact with the outer cochlear wall. To monitor the three critical cochlear walls during the insertion, a miniaturized, forward-facing OCT probe was affixed to the top of the CI insertion sheath (Fig. 1a–c). Details of the optical design and fabrication are provided in Methods. The probe is connected to the OCT system, generating tissue images, which can be displayed to the surgeon during the insertion. The optically-guided sheath is inserted into the cochlea such that its wing points towards the modiolus and the probe is facing the top wall of the ST (Fig. 1d). Integration of the OCT probe in the sheath design makes it side-specific. For the purpose of consistency and experimental convenience, only cochleae of right-sided inner ears (samples #1–8) were used in this study.

### Distance assessment during insertion

Accuracy of distance assessment with the OCT probe was investigated in a controlled experiment, whereby the optically-guided sheath was moved on a translational stage with respect to the surface of a Petri dish containing saline solution (Supplementary Fig. 1). Physiological saline solution was used to mimic the refractive index of fluid inside the cochlea. The maximum imaging depth of the OCT system was 2.7 mm (assuming a refractive index of 1.32)^35^. The translation distance calculated from the OCT images was compared to the readings of the translation stage. The accuracy of distance assessment with OCT was calculated as the difference between the measurements of distance from the OCT and from the translational stage measurement, and amounted to 0.01 ± 0.009 mm (mean ± standard deviation; n = 21 measurements).

### Identification of the cochlear walls

**Figure 2:**
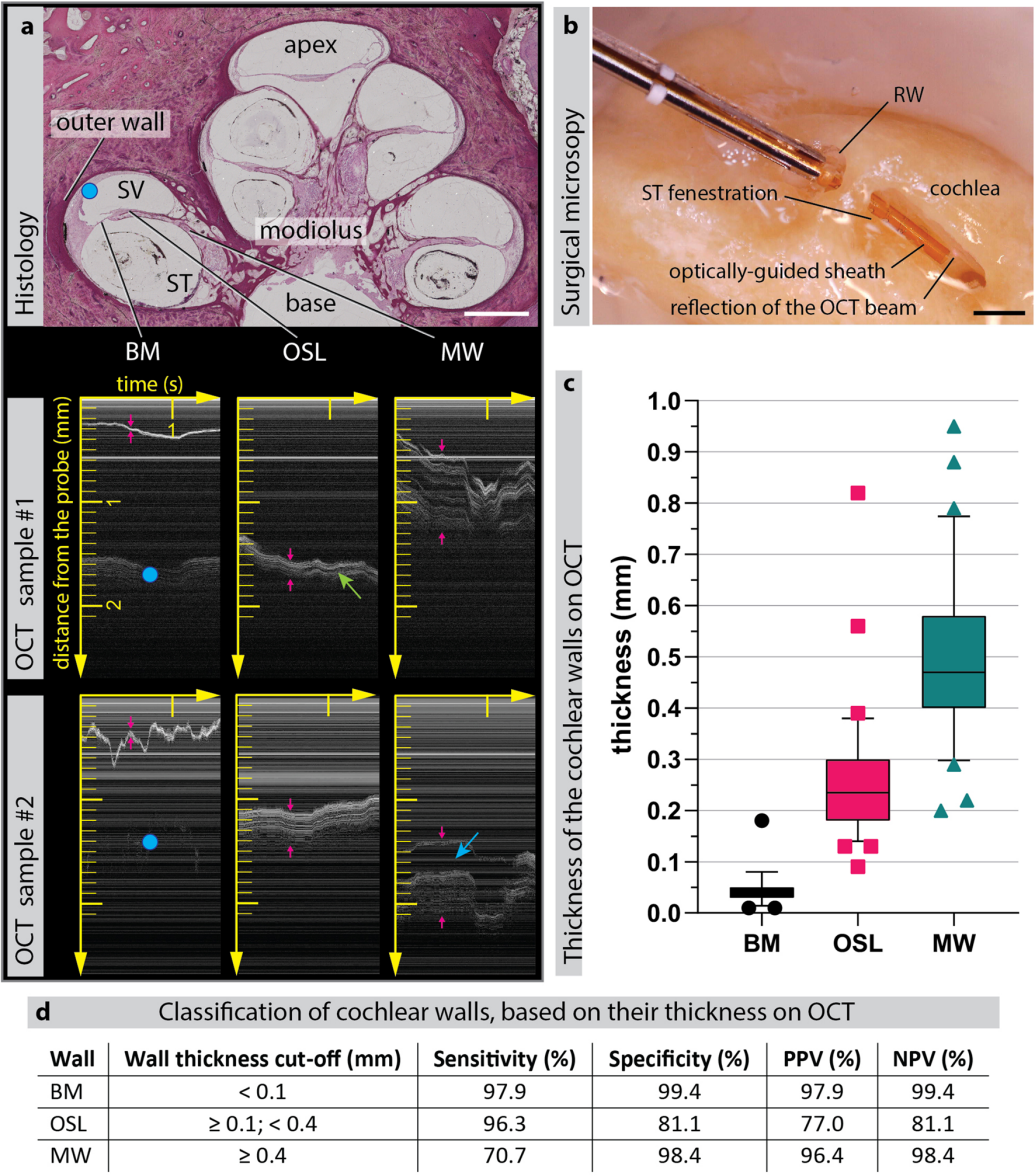
Thickness-based identification of the cochlear walls with the optically-guided sheath. (**a**) Histological section of a human cochlea is provided to illustrate the imaged cochlear walls, with indication of the cochlear apex, base, modiolus, the cochlear compartments (scala vestibuli and scala tympani) and the outer wall. Below, representative OCT M-scans of the BM, OSL and MW in samples #1 and #2 are shown, which were acquired by means of optically-guided sheath. The top of each OCT image corresponds to the position of the probe tip. Each OCT A-scan acquisition corresponds to a column of the OCT image, with a sequence of A-scans (M-scan) acquired over time as the probe is inserted and displayed left to right. The horizontal axis shows time in seconds (most recent A-scans appear on the right side of the M-scan) and the vertical axis shows distance from the probe in millimeters. Horizontal lines in OCT are due to additional back-reflections within the OCT system, which form a constant noise pattern. Red arrows indicate the thickness of the cochlear walls. The wall of the scala vestibuli can sometimes be visualized through the BM (blue dot). Green arrow indicates the gap between the two bony laminae of the OSL, visualized on OCT. Blue arrow indicates a large non-bony gap, presumably corresponding to the Rosenthal’s canal within the MW, which contains the auditory nerves. (**b**) Microscopic photograph of the optically-guided sheath inside the fenestrated ST of the sample #1, with visible reflection of the near-infrared OCT beam from the OSL cochlear wall (**c**) Box & whiskers graph, depicting OCT thickness of the three analyzed cochlear walls in samples #1 and #2 (n = 202 measurements: 47 BM, 80 OSL, 75 MW) with their respective median, interquartile range and 5–95 percentile. (**d**) The cut-offs of the thickness-based cochlear wall identification with their resulting sensitivity, specificity, positive predictive value (PPV) and negative predictive value (NPV). *BM* basilar membrane, *MW* modiolar wall, *OSL* osseous spiral lamina, *RW* round window, *ST* scala tympani, *SV* scala vestibuli. Scale bar: 1 mm.

OCT is a subsurface imaging modality, in which the OCT light beam penetrates approximately 1.5 mm into turbid tissue^36^, providing the possibility to measure wall thickness. Based on the anatomical characteristics of the three critical cochlear walls (BM, OSL, MW), we hypothesized that they can be classified, based on their thickness on the OCT scan (Fig. 2a). The cochlear walls are visualized using Motion-mode (M-mode) imaging, as a series of one-dimensional Amplitude-scans (A-scans) over time, continually being acquired as the optically-guided sheath is inserted. The A-scan is a depth-resolved one-dimensional reflectivity profile acquired along the direction of the light beam, where the amplitude at each point equals the magnitude of the OCT signal^29^. We measured the thickness of these cochlear walls on the OCT M-scans, recorded during the insertion of the optically-guided sheath into two human cochleae, samples #1 and #2. Note that, because the probe is not perpendicular to the wall during insertion into the cochlea, the apparent structure thickness on the OCT scan is larger than the actual structure thickness and depends on the incidence angle of the OCT beam. To verify which wall was imaged, the intracochlear position of the optically-guided sheath was visualized under the microscope through a fenestration in the ST (Fig. 2b).

As expected, the cochlear walls significantly differed in their apparent thickness on the OCT images, which allowed us to classify them as follows: BM is < 0.1 mm; OSL is between ≥ 0.1 mm and < 0.4 mm; MW is ≥ 0.4 mm (Fig. 2c). This classification enabled highly sensitive (97.9%) and specific identification (99.4%) of the BM (Fig. 2d). For the OSL and the MW, which had overlapping apparent thickness ranges, the cut-off value of 0.4 mm was empirically chosen to maximize the detection accuracy of the OSL (96.3%), which is most at risk of trauma^8,25,37,38^.

### Correction of insertion trajectory, based on real-time OCT feedback

**Figure 3:**
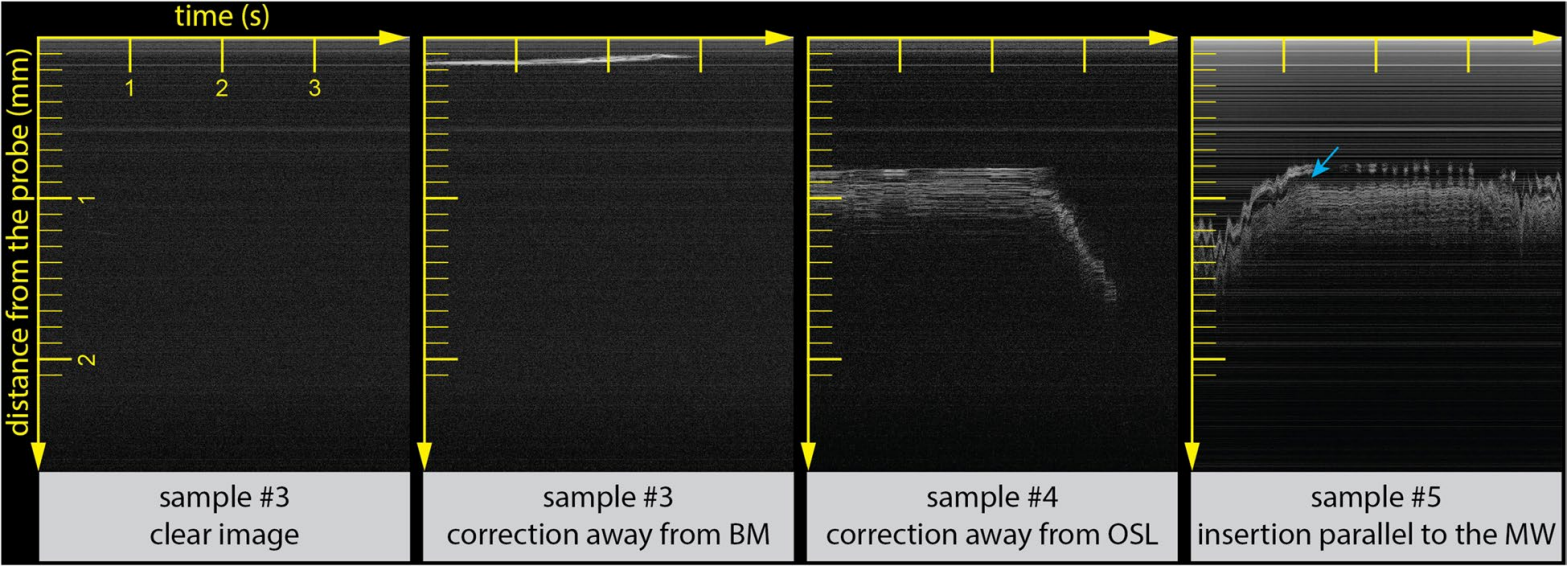
OCT-guided correction of insertion trajectory. (left) Example of a clear image, indicating a perfect insertion trajectory with no obstacles ahead for the imaging depth of the OCT probe. (middle left) Insertion parallel to the BM at a very close distance, followed by correction of the insertion trajectory, resulting in absence of visualized obstacles ahead of the optically-guided sheath. (middle right) Insertion parallel to the OSL at a relatively safe distance, followed by correction of the insertion trajectory (right side of image), resulting in increased distance and subsequent absence of visualized obstacles. (right) Insertion parallel to the MW at a relatively safe distance. Blue arrow indicates a large non-bony gap, presumably corresponding to the Rosenthal’s canal within the MW, which contains the auditory nerves. Horizontal and vertical axis are in correspondence with Fig. 2. *BM* basilar membrane; *MW* modiolar wall, *OSL* osseous spiral lamina.

Having established that the optically-guided sheath enables accurate distance assessment and identification of cochlear walls, we evaluated whether it is feasible for a CI surgeon to manually adjust the insertion trajectory, based on the OCT feedback. Three experienced CI surgeons performed insertions of the optically-guided sheath into three human cochleae, samples #3, #4 and #5. In all samples, full insertion of the sheath could be achieved using standard surgical approaches. The operators corrected the trajectory during the insertion, based on real-time OCT feedback, if they deemed it necessary to avoid contact between the cochlear walls and the optically-guided sheath. Trajectory correction resulted in an increased distance between the probe and the cochlear wall on the OCT image, or a complete disappearance of the cochlear wall from the OCT imaging range. A clear image without visualization of any cochlear walls was interpreted as a perfect insertion trajectory (Fig. 3).

### Wall contact during insertion as an indicator of trauma

**Figure 4:**
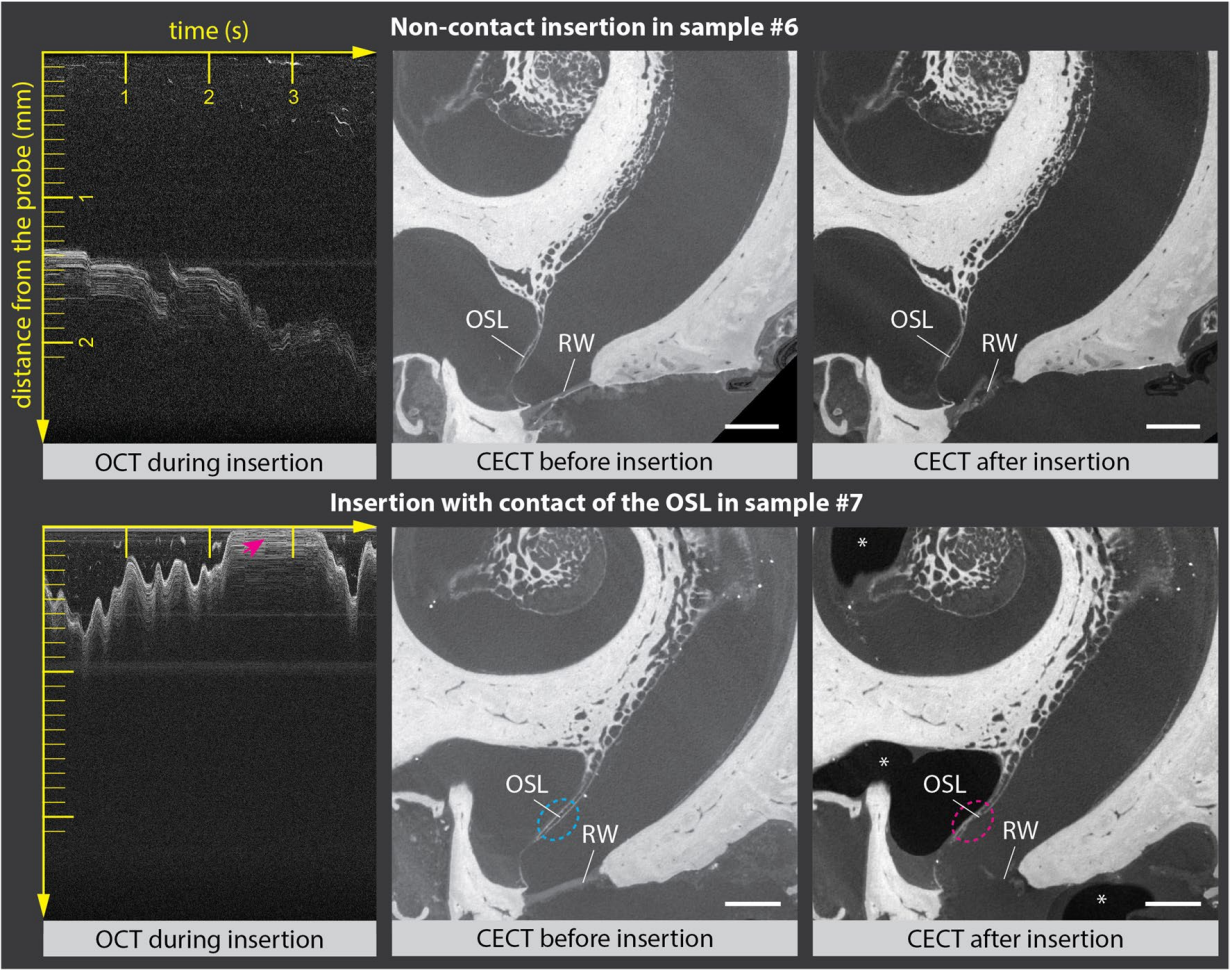
Wall contact during insertion as an indicator of trauma. (top) Experimental data of sample #6: intra-insertion OCT data, showing no contact between the optically-guided sheath and the OSL, together with the pre- and post-insertion CECT images, showing no trauma. (bottom) Experimental data of sample #7: intra-insertion OCT data together with the pre- and post-insertion CECT images. The blue ellipse indicates intact OSL on pre-insertion CECT images. The red arrow shows contact between the optically-guided sheath and the OSL, detectable on real-time OCT images, and the resulting impression microtrauma of the OSL (red ellipse) on CECT images, acquired after the insertion. On the post-insertion CECT image, (*) indicates the presence of intracochlear air, which was not related to the insertion. In both cochleae, the optically-guided sheath was inserted through an incision in the membrane, covering the round window. Horizontal and vertical axis of the OCT images are in correspondence with Fig. 2. *OSL*, osseous spiral lamina, *RW* round window. Scale bar: 1 mm.

Since our method for avoiding trauma relies on non-contact insertion of the optically-guided sheath into the cochlea, we investigated whether an absence of visualized contact between the probe and the cochlear walls was indeed related to the avoidance of trauma. We performed CECT-controlled experiments for guided insertion of the optically-guided sheath into two human cochleae. In sample #6, the optically-guided sheath was inserted in a non-contact mode. In the sample #7, a brief contact between the optically-guided sheath and the visualized OSL was allowed. CECT-images showed no trauma in sample #6 (Fig. 4, top), and a small impression trauma of the OSL in sample #7 (Fig. 4, bottom).

### Insertion of the slim modiolar electrode through the optically-guided sheath

**Figure 5:**
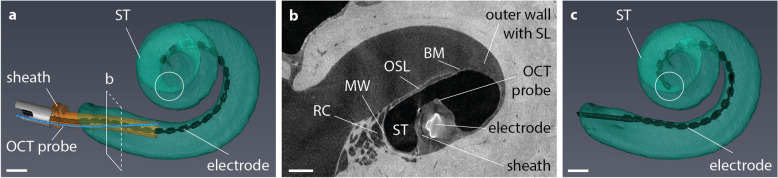
Intracochlear position of the optically-guided sheath and the slim modiolar electrode. (**a**) CECT-based 3D rendering of the optically-guided sheath together with the slim modiolar electrode inside the ST in sample #8. The rectangle indicates the position of the CECT section in (**b**). Circle indicates the final position of the electrode array tip, as shown in (**c**). (**b**) CECT cross-section showing the position of the optically-guided sheath within the ST with respect to the three critical cochlear walls. (**c**) CECT-based 3D rendering of the final electrode position inside the ST, after extraction of the optically-guided sheath. Note that all electrode contacts are perfectly positioned inside the ST in close proximity to the modiolar wall. The position of the electrode array tip is encircled. *BM* basilar membrane, *MW* modiolar wall, *OSL* osseous spiral lamina, *RC* Rosenthal’s canal, containing auditory nerve fibers, *ST* scala tympani. Scale bar: 1 mm.

Finally, we tested whether the modification of the sheath affects the final position of the slim modiolar electrode itself. We performed an insertion of the optically-guided sheath, together with the preloaded electrode in a separate sample #8, which was immediately followed by the insertion of the electrode array. CECT imaging was performed after the insertion of the electrode array and after the subsequent extraction of the optically-guided sheath. CECT-based 3D renderings demonstrated full insertion of the slim modiolar electrode in perimodiolar position without any evidence of translocation or tip fold-over (Fig. 5).

## Discussion

The objective of this study was to enable intra-operative guidance of insertion of a perimodiolar electrode sheath into the human cochlea, by integrating a miniaturized forward-facing OCT probe. In a series of experiments, conducted on real human cochleae, we demonstrated that the integrated OCT functionality enables the surgeon to monitor the relative position of the optically-guided sheath within the cochlear lumen in real-time during the insertion. Based on OCT feedback, the surgeon was able to adjust the orientation of the insertion so that the sheath does not come in contact with the cochlear walls, reducing the risk of trauma. Integration of the OCT probe into the sheath design did not significantly affect the insertion mechanics: the slim modiolar electrode could be fully inserted into the cochlea through the optically-guided sheath in perimodiolar position without evidence of translocation or tip fold-over.

The position and orientation of the sheath inside the ST plays a central role in the insertion of the slim modiolar electrode. Whereas optimal sheath position almost guarantees a perfect electrode insertion, incorrect placement of the sheath can result in electrode translocation and electrode tip fold-over^13–21^. Both of these complications pose a high risk for structural damage leading to residual hearing loss and suboptimal CI performance^13–21^. Additionally, several studies reported difficulty and increased resistance when inserting the sheath through the round window in some patients, which could only be improved with extensive thinning of the cochlear bone above the round window (bony overhang)^16,17,39^. During our experiments, we perceived that in such cases, the sheath typically heads towards the proximal vertical part of the OSL. In conventional blind insertion, the insertion trajectory is adjusted upon resistance, but this does not prevent the initial contact between the sheath and the cochlear wall, which can traumatize the OSL. Such trauma is too small to be detected with current clinical imaging techniques (CT and MRI)^5,40^, but could negatively affect the residual hearing. The thin auditory nerve fibers running through the OSL can be damaged, and any structural trauma—especially in presence of loose bone debris—can trigger generalized inflammation and thus fibrosis or ossification of the cochlear lumen^4^.

The results of this study demonstrated, for the first time, that the optically-guided sheath enables visualization of the cochlear walls, allowing the surgeon to adjust the insertion trajectory to improve the positioning of the sheath within the ST and avoid insertion trauma. In practice, obstacles, such as cochlear walls, could be detected across 1.5–2 mm of the lumen. Whilst the OCT system could detect signal up to 2.7 mm, the useful imaging distance is reduced by divergence of the OCT light beam, which tends to be more limited with highly miniaturized optics such as those used in our probe^36^. Distance from the probe could be assessed with an accuracy of ± 0.01 mm. Given that the electrode insertion speed for manual electrode insertion is approximately 0.87 mm/s^41^, the OCT feedback gives the surgeon sufficient imaging distance for timely adjustment of the insertion trajectory. The feasibility of manual trajectory adjustment was also confirmed in OCT-guided insertion experiments on real human cochleae, conducted by three experienced CI surgeons.

In recent years, advances have been made towards robotic insertion of cochlear implants, as it may provide improvements in control of the speed and direction of the insertion necessary to preserve residual hearing^42^. Torres et al.^28^ presented a system that calculated the insertion trajectory based on the shape of the ST extracted from pre-operative CT images. Despite promising results, CT-based alignment still cannot fully prevent the trauma of the BM^28^, which poses a high risk for the residual hearing function^43^. The incorporation of an OCT probe into the robotic insertion setup could greatly improve the outcomes, enabling real-time monitoring of the cochlear lumen during the insertion. In particular, OCT imaging enables excellent identification of the BM with sensitivity and specificity > 97%.

Aside from visualizing normal cochlear walls, OCT imaging has the potential to detect other obstacles on the insertion path, such as pathological fibrosis and ossification of the ST. This is particularly important in patients who have previously undergone CI surgery^44,45^, in patients with cochlear otosclerosis^46^ and when cochlear hearing loss is related to a meningitis infection^47^. In patients with these pathologies, the outcome can often be complicated by incomplete electrode insertions, electrode translocations and device failures^46^. Furthermore, the cochlear obstruction is often not clearly visible on preoperative imaging, increasing the risk of complications^47^. OCT-guided insertion could help the surgeon estimate the risk of obstruction intraoperatively and decide whether the obstacle can be bypassed by adjusting the insertion trajectory or if an alternative insertion route is necessary (e.g. scala vestibuli insertion).

In this study, we integrated one forward-facing, single-fiber OCT probe into the sheath of the slim modiolar electrode. The incorporation of multiple single-fiber OCT probes is feasible and would provide greater imaging coverage. In a similar way, one or multiple probes could also be integrated into the electrode array itself, to allow OCT monitoring of the insertion into the deeper cochlear regions. Additionally, integration of optical fibers into the electrode array offers the possibility of incorporating force sensors through the use of fiber Bragg gratings (FBG)^48–52^. This could provide real-time feedback of the forces that lead to trauma. Early work has demonstrated the feasibility on combining OCT and FBG into a single device^53^. In parallel work by other groups, insertion of optical fibers into the cochlea is being explored in the context of the development of an optical CI^54^ to replace the current electrode-based CI.

In addition to CIs, OCT technology could have applications in the fields of inner ear diagnostics and regenerative therapies^55^. Regenerative therapies, relying on mesenchymal stem cells and adeno-associated viral inner-ear gene transfer, may need to be injected into the scala media compartment of the cochlea^56,57^. To enable optogenetic stimulation in optical CIs, photosensitivity of the auditory neurons has to be induced by injection of an adeno-associated virus-mediated gene^58^. There is currently no method to safely insert diagnostic or therapeutic devices into the specific area of cochlea, without causing trauma. OCT-guided insertion could aid in the insertion of the injection device, such as a microneedle^55^, into the scala media or into the Rosenthal’s canal, containing the auditory neurons, from within the ST, while at the same time avoiding unnecessary trauma of the other structures. A proof-of-concept device has previously been demonstrated that integrates an OCT probe with a needle capable of injecting fluid^59^. For the purpose of intracochlear diagnostics, OCT technology can not only be used for atraumatic insertion of the device, but also for structural evaluation by means of high-resolution imaging ^32^. For example, a forward-facing OCT probe for guided insertion could be combined with a rotating side-facing OCT probe capable of acquiring a 3D tomographic image of the cochlear lumen prior to other surgical interventions or for intracochlear diagnostics.

In summary, the integration of fiber-optic OCT probes offers the potential to enable highly accurate, guided insertion of cochlear implants, regenerative inner ear therapies and devices for intracochlear diagnostics. Contrary to standard clinical imaging techniques, which remain limited in their resolution, OCT enables rapid subsurface imaging of tissues at a resolution of just a few micrometers. The safety of OCT technology in living patients has been demonstrated by numerous applications in different domains of clinical medicine^30^. OCT imaging is also already actively being used in clinical otology for middle ear diagnostics^60^, including several intraoperative applications^61,62^. Functional evaluation by means of vibrometry OCT can have added value for inner ear diagnostics in addition to the high-resolution morphological evaluation^60^. The OCT probes can also be sterilized for intraoperative use and their relatively low cost allows for the probes to be discarded after each surgery^63^.

OCT imaging technology does have some inherent limitations. The primary disadvantage is that infrared light has a limited penetration depth in scattering biological tissues^30^, typically 1–1.5 mm^36,64^, which prohibits external scanning of the cochlea. However, this is addressed through the use of highly-miniaturized probes that are deployed inside the cochlea, as proposed in our study. The clear intracochlear fluid allows visualization of obstacles well ahead of the probe, giving the CI surgeon sufficient time to adjust the insertion direction, if deemed necessary. Integration of fiberoptic OCT probes into the CI will also increase the diameter of the inserted device. In the future, this may be addressed by more intimately embedding and encapsulating the probe inside the insertion sheath. This approach would also provide additional robustness for the OCT glass fiber, reducing the risk of fiber breakage during insertion. The integration of optical fibers is also likely to make the sheath more rigid, which could increase the risk of trauma if non-contact insertion would not be possible in some cochleae^13^. There may be potential to improve the flexibility of the OCT fiber by reducing its diameter^65^ or through the use of polymer^66^ or soft glass^67^ fibers, although this has not been explored in this paper. Finally, we noticed that whereas OCT guidance provides valuable visual cues, the mechanical feedback—the surgically experienced resistance—still plays and important role in the interpretation of the insertion trajectory. These findings motivate further quantitative systematic research into mechanical insertion forces underlying trauma of the different intracochlear structures, which are poorly understood^26,68^, with the purpose of improving the surgical techniques and electrode designs for atraumatic insertion.

In conclusion, our optically-guided sheath has the potential to provide improved intra-operative guidance of cochlear implants during insertion, which would have particular benefit in reducing risk of complications for patients with residual hearing. This paper introduced a prototype device and has validated its use through ex vivo human cochlear experiments, establishing the ability of OCT to quantify distances to the cochlear wall with high accuracy and to distinguish between three critical cochlear walls (basilar membrane, osseous spiral lamina, modiolar wall) with high sensitivity and specificity. As such, the presented results set the stage for further clinical trials to evaluate its safety and efficacy in cochlear implant patients, and warrant further research into thinner, more flexible optical fibers, which could be incorporated into the electrode array itself to enable real-time monitoring of the insertion path throughout the entire human cochlea.

## Methods

### Human cochleae

Eight right-sided fresh-frozen human cadaveric inner ears (samples #1-8) were used in this study, as it is the common practice for electrode insertion studies^13^. All samples were harvested within 72 h post mortem from individuals who underwent a clinical brain autopsy at the University Hospitals of Leuven. Informed consent was obtained from all subjects, their next of kin or legal guardian(s). Harvesting and use of the temporal bones was conducted in accordance with the Helsinki Declaration and approved by the Medical Ethics Committee of the University Hospitals of Leuven (S65502). To optimize the accessibility of the cochlea for the experiments, the inner ears (approx. 10 mm × 10 mm × 20 mm) were dissected out of the temporal bones in accordance with the previously described methodology^69^. In the samples #1 and #2, the outer wall of the ST was carefully fenestrated with the surgical diamond drill of 1.0–1.5 mm, to directly visualize the intracochlear space. This way, the position of the probe with respect to the cochlear walls of the ST could be visualized under the microscope, simultaneously with the real-time OCT imaging. The samples were not fixed or decalcified. If it was necessary to store the samples up to one week between different experiments, they were preserved in the refrigerator at 4 °C. If longer storage was necessary, the samples were frozen at − 20 °C and thawed overnight at 4 °C on the evening before the experiment. No substantial change in the appearance of the studied intracochlear structures could be detected with OCT in consequence of an additional freeze-thawing cycle. During the first insertion, the cochleae contained the original perilymph. For multiple insertions, the cochleae were refilled with saline solution.

### Fabrication of the optically-guided sheath

The forward-facing OCT probe consists of a length of single-mode optical fiber (SMF 28, Thorlabs Inc., Newton, NJ, USA) with miniaturized focusing optics fabricated on the distal end to form a weakly-focused light beam (i.e. low numerical aperture) with a beam waist of approximately 20 μm. In detail, the single-mode fiber is terminated with a 350 µm length of no-core fiber (NCF125, Success Prime Corporation, Miaoli County, Taiwan) which enables the light beam to expand, and 150 µm of graded-index (GRIN) fiber (DrakaElite 100/125 µm, Drake Communications Inc., Krum, TX, USA), which subsequently focuses the expanded light beam (Fig. 1c). The distal end of the GRIN fiber is coated with a thin layer of silver such that 8% of the light is reflected to form a reference signal for this common-path OCT setup, whilst the remainder of the light is emitted into the tissue for imaging. The silver coating of the GRIN fiber was performed using a bespoke evaporative metal coating setup. The thickness of the silver coating is set so that the reference signal does not saturate during detection of the OCT signal. The stripped single-mode optical fiber was adhered externally on top of the sheath of slim modiolar electrode (Nucleus^®^ CI532/CI632; Cochlear Ltd., Sydney, Australia) using cyanoacrylate glue. This position was chosen to provide imaging of the cochlear walls most at risk of trauma during insertion (OSL, BM).

### OCT imaging and processing

The fiber-optic probe of the optically-guided sheath was connected to a spectral-domain OCT system (Telesto TEL321C1; Thorlabs, Lübeck, Germany) with the following characteristics: central wavelength of 1300 nm and axial resolution of 4.2 μm in water. We note that the image penetration depth in tissue of OCT is limited by optical scattering, not absorption, and that the scattering coefficient of tissue reduces at longer wavelengths. Whilst alternative OCT systems are available at a range of shorter wavelengths, a central wavelength of 1300 nm was chosen to maximize the image penetration depth^29^. Each measurement acquired with the OCT system consisted of a 1-D depth scan (referred to as an A-scan) of the area immediately in front of the fiber. The width of this measurement is specified by the diameter of the light beam. For our device, the beam waist was approximately 20 μm. Depending on the proportion of the near-infrared light that was reflected by the silver-coating at the distal tip of each OCT probe, imaging was performed at the rate of 76 kHz or 146 kHz so as to avoid saturating the detector, whereby each image point of the M-scan was calculated as an average of 5 successive A-scans. For intracochlear imaging, the wavelength-specific refractive index of water (1.32) was applied^35^, which is considered equivalent to the perilymph and the saline solution^70^. During the insertion, OCT data were acquired and viewed in real-time in ThorImage software (ThorLabs, Lübeck, Germany), using M-mode visualization (a sequence of A-scans displayed over time). The signal range was adjusted to the dynamic range of the visualized intracochlear structures. When the structures of interest were visualized, snapshots of the M-mode window were saved.

Each optically-guided sheath was re-used whilst the OCT fiber remained intact and connected to the sheath, which was between 30 and 300 insertions. With the inclusion of initial feasibility experiments, a total number of 6 optically-guided sheaths was used in this study for over 1300 insertions. Intracochlear breakage of the fiber tip occurred twice during surgical insertion training and 3 times during the validation experiments for identification of the cochlear walls.

### OCT-guided insertion

To insert the optically-guided sheath into the cochlea, the sheath was manually operated with handheld Hartmann tweezers. During the insertion, the surgeons were intermittently looking either at the surgical field through the microscope or at the OCT images on the screen of the OCT system. All insertions except for sample #5, were performed through the round window. If necessary, the round window opening was extended to improve the positioning of the probe. In sample #5, the optically-guided sheath was inserted through a surgically drilled opening adjacent to the round window, the cochleostomy. The assessment of any mechanical resistance experienced during insertion was based on the judgement of the CI surgeons.

### CECT imaging and processing

Contrast-enhanced microCT imaging (CECT) was used to evaluate whether OCT-guided insertion resulted in intracochlear trauma (in samples #6 and #7) and to determine the final position of the optically-guided sheath and the slim modiolar electrode within the cochlea (in sample #8). The cochleae were placed on a gentle shaker for 5–7 days, while they were submersed in Hafnium-substituted Wells–Dawson polyoxometalate (K_16_[Hf(α_2_-P_2_W_17_O_61_)_2_]·19H_2_O) in phosphate-buffered saline solution^71^. CECT data were acquired prior to and after the insertion. In samples #6 and #7, the optically-guided sheath was extracted before CECT imaging. In sample #8, the fiber of the OCT probe was cut after the guided insertion and the inserted electrode was fixated in place with dental wax and parafilm foil. Then, CECT imaging was carried out when both the optically-guided sheath and the electrode were inside the cochlea and after the extraction of the sheath while the electrode remained within the cochlea, in accordance to the standard procedure for insertion of the slim modiolar electrode^13^.

The cochleae were imaged using a Phoenix Nanotom M (GE Sensing & Inspection Technologies GmbH, Wunstorf, Germany), equipped with a tungsten target, at 6.3 µm isotropic voxel size. 2400 frames were acquired over 360°.

Without the electrode, the microCT device was operated at a voltage of 50 kV and a current of 531 µA, with exposure time of 500 ms, without a filter; the dataset of the sample with an electrode was acquired at a voltage of 100 kV and a current of 265 µA, with exposure time of 750 ms whereby a platinum-gold-coated 1.0 mm filter of aluminum was used to reduce the beam hardening artifacts. The data were reconstructed in Datos|x (GE Sensing & Inspection Technologies GmbH, Wunstorf, Germany), while applying scan optimization (projection filter, inline volume filter, and beam hardening correction). The files were exported as 16-bit .tiff slices and converted to .jpeg images, whereby histogram window was automatically adjusted to the dynamic range of the dataset using an in-house developed MatLab tool (MathWorks, MA, USA)^72^.

On the data of sample #8 with electrode, the metal artefacts were suppressed by applying a simple projection completion approach using linear interpolation^73^. For that purpose, an initial reconstruction was made with the Feldkamp-Davis-Kress (FDK) algorithm^74^. In that reconstruction, the metals were segmented using thresholding. The resulting binary metal image was forward projected to identify in the projections all pixels that had been affected by high metal attenuation. The values of those pixels were replaced with interpolated values, computed from the neighboring pixel values that were not affected by metals. The final image was computed from the corrected projections with FDK.

CECT data were cropped to the cochlear region in CTAn (Bruker MicroCT, Kontich, Belgium) and reoriented in accordance with the cochlear coordinate system^75^ in DataViewer (Bruker MicroCT, Kontich, Belgium). 3D renderings of sample #8 were generated based on thresholding and manual segmentation in Avizo (FEI Visualization Sciences Group, Thermo Fisher Scientific Inc., Bordeaux, France). Prior to the segmentation, the datasets were resized to an isometric voxel size of 18.9 µm^3^ in CTAn.

### Histological analysis

A separate cochlea sample was sent for histological analysis. This sample was fixed in a 4% formaldehyde solution for 5 days, dehydrated in ethanol 50% and 70% and imaged using standard microCT to guide the position of the 2D histological sections. LLS Rowiak (LaserLabSolutions, Hanover, Germany) performed polymethylmethacrylate embedding, OCT-guided sectioning with a laser microtome TissueSurgeon^76^ and staining of the slices with eosin-hematoxylin.

### Data analysis

Data were stored, analyzed and graphically visualized in Excel. The significance of thickness difference between the three cochlear walls was determined by applying non-paired, one-way student *t* test, whereby the outcome of p ≤ 0.05 was interpreted as significant.

## Supplementary Information

Supplementary Figure 1.

## Data Availability

All data analyzed during this study are included in this published article. The full OCT and CECT datasets can be provided by Nicolas Verhaert (nicolas.verhaert@kuleuven.be) upon reasonable request.
