# Supplementary material for: An optically-guided cochlear implant sheath for real-time monitoring of electrode insertion into the human cochlea
Source: Sci Rep. 2022 Nov 10;12:19234. doi: 10.1038/s41598-022-23653-4 (PMC9649659; doi:10.1038/s41598-022-23653-4)
Supplement: Supplementary file 1 — Supplementary Figure 1. [file 41598_2022_23653_MOESM1_ESM.pdf]

Supplementary information for

An optically-guided cochlear implant sheath for real-time monitoring of electrode insertion into the human cochlea

Starovoyt Anastasiya [1, 2, *], *Quirk Bryden C.* [3, 4], *Putzeys Tristan* [1, 2, 5], *Kerckhofs Greet* [6, 7, 8, 9], *Nuyts Johan* [10, 11], *Wouters Jan* [1, 2], *McLaughlin Robert A.* [3, 4, 12, +], *Verhaert Nicolas* [1, 2, 13, +]

Author affiliations:

1. ExpORL, Department of Neurosciences, KU Leuven, 3000 Leuven, Belgium
2. Leuven Brain Institute, Department of Neurosciences, KU Leuven, 3000 Leuven, Belgium
3. Australian Research Council Centre of Excellence for Nanoscale BioPhotonics, Faculty of Health and Medical Sciences, The University of Adelaide, Adelaide, SA, 5005, Australia
4. Institute for Photonics and Advanced Sensing, The University of Adelaide, Adelaide, SA, 5005, Australia
5. Laboratory for Soft Matter and Biophysics, Department of Physics and Astronomy, KU Leuven, 3000 Leuven, Belgium
6. Biomechanics Laboratory, Institute of Mechanics, Materials, and Civil Engineering, UCLouvain, 1348 Louvain-la-Neuve, Belgium
7. Department of Materials Science and Engineering, KU Leuven, 3000 Leuven, Belgium
8. Institute of Experimental and Clinical Research, UCLouvain, 1200 Woluwé-Saint-Lambert, Belgium
9. Prometheus, Division of Skeletal Tissue Engineering, KU Leuven, 3000 Leuven, Belgium
10. Department of Imaging and Pathology, Division of Nuclear Medicine, KU Leuven, 3000 Leuven, Belgium.
11. Nuclear Medicine and Molecular Imaging, Medical Imaging Research Center, 3000 Leuven, Belgium
12. School of Engineering, University of Western Australia, Perth, WA, 6009, Australia
13. Dept. of Otorhinolaryngology, Head and Neck Surgery, University Hospitals of Leuven, 3000 Leuven, Belgium

*Corresponding author: Anastasiya Starovoyt (email: anastasiya.starovoyt@kuleuven.be)
+ these authors contributed equally to this work and share last authorship.

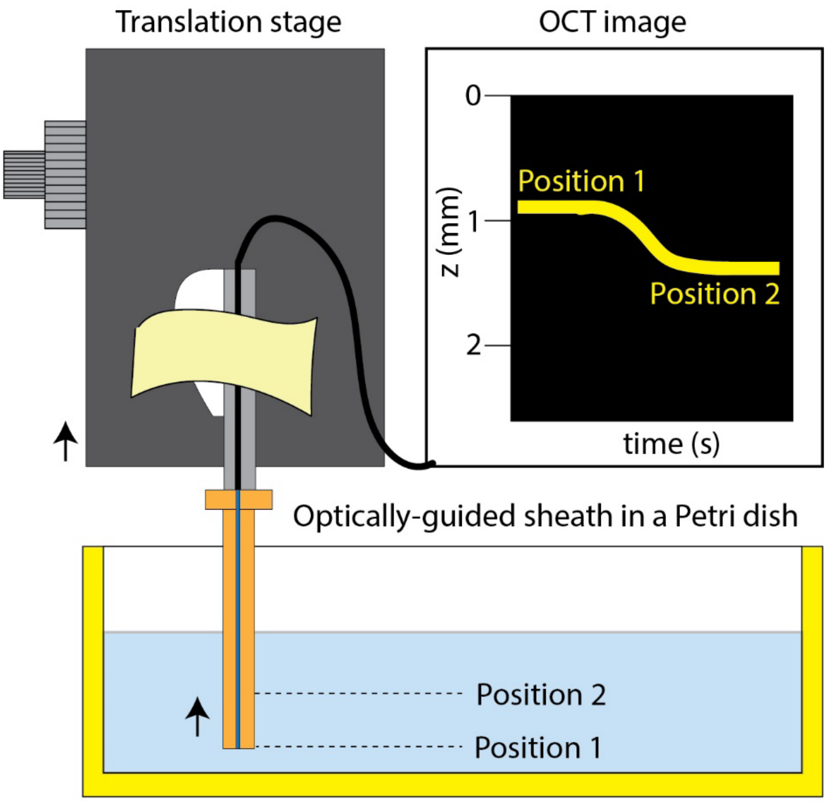

Calculated difference between measurements

	OCT image	Translational stage
Position 1 (mm)	0.85	2.31
Position 2 (mm)	1.4	2.85
Distance (mm)	0.55	0.54
Δ OCT – stage (mm)		0.01

Supplementary Figure 1. Measurement of the accuracy of distance assessment with the OCT probe. The optically-guided sheath was taped onto a translation stage, whereby the OCT probe tip was submersed in saline solution, contained inside a Petri dish. During the experiment, the sheath was moved up and down, while OCT images of the Petri dish surface were acquired in real-time. The distance between the OCT probe tip and the surface of the Petri dish was recorded at different positions, together with the corresponding stand of the translation stage. An example of the recorded values is shown in the table. The accuracy of distance assessment with the OCT probe was calculated based on n = 21 translations.
